# Hispidulin Enhances Temozolomide (TMZ)-Induced Cytotoxicity against Malignant Glioma Cells *In Vitro* by Inhibiting Autophagy

**DOI:** 10.1155/2022/5266770

**Published:** 2022-06-28

**Authors:** Zhihua Chen, Guofeng Zhu, Chunpeng Sheng, Jianwei Lei, Sihui Song, Jianming Zhu

**Affiliations:** Department of Neurosurgery, The Second Affiliated Hospital of Nanchang University, Nanchang 330006, China

## Abstract

Temozolomide (TMZ), an oral alkylating agent, is the widely used first-line chemotherapeutic reagent for glioma in clinical practice. However, TMZ-induced autophagy is another cellular process favoring glioma cell survival. This study aimed to explore whether hispidulin can facilitate TMZ-induced cell death of glioma. The MTT assay showed that coadministration with hispidulin and TMZ could significantly decrease the viability of glioma U87MG cells. Meanwhile, hispidulin administration was also observed to promote TMZ-induced apoptosis. Furthermore, additional hispidulin treatment further elevated TMZ-induced expression of Bax, cleaved-caspase-9, and cleaved-caspase-3 protein but decreased Bcl-2 protein expression in U87MG cells. We also observed that hispidulin suppressed TMZ-induced autophagy to promote apoptosis, as showed by decreased AVOs and LC3B-I/II protein expression. These results collectively suggested that the combination of hispidulin and TMZ could improve the antitumor efficiency of TMZ against malignant gliomas.

## 1. Introduction

Glioma is the most lethal cancer that arises from the central nervous system (CNS) in adults [1, 2]. Despite enormous progress in surgical resection, radiotherapy, and chemotherapy, the prognosis is still disappointing [3]. The World Health Organization (WHO) has declared that more than one-third of patients with glioma could be prevented [4]. In 2000, temozolomide (TMZ) has received approval as the first-line chemotherapeutic drug for malignant glioma due to its easy absorption and penetration through the blood-brain barrier [5, 6]. However, increasing evidence has demonstrated a relative resistance to TMZ is detected in most malignant glioma cases [7]. Indeed, TMZ treatment induces glioma cell autophagy and thereby favors glioma cell survival. Furthermore, recent studies have suggested that the autophagy inhibitors are able to enhance TMZ-induced apoptosis in glioma cells [8, 9]. Consequently, searching for novel adjuvant chemotherapeutic agents has become especially highlighted.

In this field, naturally occurring chemical compounds derived from plants are gaining much attention as chemopreventive agents for cancers. Hispidulin (4′,5,7-trihydroxy-6-methoxyflavone), the most studied flavonoid, presents in different plants, such as Salvia involucrata, Crossostephium Chinese, and Arrabidaea chica [10] and displays a wide range of pharmacological activities, including antifungal, anticonvulsant, antiepileptic, anti-inflammatory, antioxidant, antithrombotic, anticancer activities, and so on [11, 12]. Moreover, a large number of reports have shown that hispidulin exerts diverse antitumor activities on gallbladder cancer, hepatocellular carcinoma, acute myeloid leukemia, renal cell carcinoma, and colorectal cancer [13]. Meanwhile, a pharmacokinetic study has demonstrated that hispidulin is easily prone to penetrate the blood-brain barrier [14]. Its antitumor effect on glioma is also reported [15, 16]. However, the role of hispidulin in glioblastoma remained mysterious. Therefore, this study is conducted to evaluate the adjuvant activity of hispidulin on TMZ-induced apoptosis and autophagy in glioma cells.

## 2. Materials and Methods

### 2.1. Materials and Chemicals

Hispidulin was purchased from Aladdin Bio-Chem Technology Co., Ltd (Shanghai, China) and dissolved in dimethyl sulfoxide (DMSO) (0.1%). Bax, cleaved-caspase-3, cleaved-caspase-9, LC3B-I, LC3B-II, and *β*-actin were from Santa Cruz Biotechnology (Santa Cruz, CA, USA). DAPI, DMSO, Dulbecco's modified Eagle medium (DMEM), and fetal bovine serum (FBS) were obtained from Sigma-Aldrich (St. Louis, MO, USA). Annexin V-fluorescein isothiocyanate (FITC)/propidium iodide (PI) kit and terminal deoxynucleotidyl transferase-meditated dUTP-fluorescein nick end labeling (TUNEL) in situ cell death detection kit were from Roche Diagnostics (Indianapolis, IN, USA). The bicinchoninic acid (BCA) protein assay kit was from Thermo Fisher Scientific, Waltham, MA, USA.

### 2.2. Cell Lines and Cell Culture

The human malignant glioma U87MG cell lines (Cell Bank of the Chinese Academy of Sciences, Shanghai, China) were maintained and routinely passaged in DMEM supplemented with 10% FBS, penicillin (100 U/mL), and streptomycin (100 U/mL) in a 5% CO_2_-humidified incubator at 37°C.

### 2.3. Cell Viability Analysis

The cytotoxic activity was assessed using a 3-(4,5-dimethylthiazol-2-yl) 2, 5-diphenyl tetrazolium bromide (MTT) assay. U87MG cells were seeded into each well at a density of 2 × 10^5^ cells/mL in a 96-well microplate. After 24 h, cells were suspended in the medium containing different concentrations of hispidulin (5, 10, 20, 40, and 100 *μ*g/ml), TMZ (50, 100, 200, 400, and 800 *μ*M), or their combination TMZ (100 *μ*g/ml of TMZ and 100 *μ*g/ml of hispidulin) for 48 h. Then, the cells were washed with PBS, and 10 *μ*L MTT (5 mg/ml) was added to each well. Following 4 h incubation at 37°C, the supernatant was discarded, and the formed formazan crystals in viable cells were lysed by adding DMSO (100 *μ*l) in each well. Each aliquot of the sample was transferred to 96-well plates, and the absorbance was measured at 570 nm using a microplate spectrophotometer.

### 2.4. TUNEL/DAPI Staining

Apoptotic cells in U87MG cells were analyzed by the In Situ Cell Death Detection Kit and DAPI as per the manufacturer's instructions. U87MG cells were cultured and seeded onto coverslips at 1 × 10^5^ cells/wells. After hispidulin treatment, the cells were washed twice with phosphate-buffered saline (PBS), fixed with 4% paraformaldehyde for 15 min at 37°C, followed by the addition of 0.1% Triton-X 100 for 5 min at 4°C. Afterward, the TUNEL reagent (30 *μ*l) was added to the cells and incubated for 2 h in the dark. Then, the cells were mixed with 1 *μ*l DAPI (1 *μ*g/ml) at room temperature for 5 min to mark cellular nuclei. Finally, the stained cells were moved to slides and visualized under a fluorescent microscope (Olympus Corp., Tokyo, Japan) at 200 × magnification. The apoptosis rate (%) was expressed as TUNEL-positive cells/total number of cells×100 based on three independent experiments (visualized in five randomly chosen fields).

### 2.5. Detection of Apoptosis

Apoptosis was evaluated using an annexin V-fluorescein isothiocyanate (FITC)/propidium iodide (PI) apoptosis detection kit according to the manufacturer's protocol. In brief, treated cells were subjected to trypsinization with 0.30% trypsin and centrifugation at 1000 g for 10 min. The cell pellets were washed with PBS, resuspended in 200 *μ*l binding buffer containing annexin V-FITC (10 *μ*g/mL) and PI (5 *μ*g/mL) each of 5 *μ*l for 15 min in the dark at room temperature, and immediately analyzed by the FACSCalibur flow cytometer and CellQuest software (Becton Dickinson, Franklin Lakes, NJ, USA) to analyze the apoptotic cells.

### 2.6. Detection of Acidic Vesicular Organelles (AVOs)

The appearance of AVOs is a direct result of autophagy, and flow cytometry was performed with acridine orange staining to detect and quantify the AVOs. In brief, the control or hispidulin-treated cells were stained with acridine orange (1 *μ*g/ml) for 15 min at room temperature. Then, cells were collected, washed, and suspended in the phenol red-free growth medium, followed by immediate fluorescence examination at 510–530 nm (green) and 4650 nm (red) using a FACSCalibur flow cytometer with CellQuest software (Becton Dickinson, San Jose, CA). Cells containing AVOs were identified as double-positive cells.

### 2.7. Western Blot Analysis

The treated or untreated cells were immediately lysed in the radioimmunoprecipitation assay (RIPA) lysis buffer containing protease inhibitors and then subjected to centrifugation at (12,000 g, 5 min). The supernatant was collected, and the protein concentration was measured with the BCA protein assay kit. Each sample with an equal amount of total protein (20 *μ*g) was electrophoresed by 10% SDS-polyacrylamide gel electrophoresis (SDS-PAGE), transferred to polyvinylidene fluoride (PVDF) membrane (Millipore, Billerica, MA, USA), blocked with 5% skim milk, and incubated at 4°C overnight with the primary antibody against Bax (1 : 1,000), Bcl-2 (1 : 1,000), cleaved caspase-3 (1 : 1,000), LC3B-I (1 : 1,000), LC3B-II (1 : 1,000), and *β*-Actin (1 : 1,000). The membranes were subsequently probed with the horseradish peroxidase-conjugated secondary antibody (1 : 5000) for 1 h at 37°C. The blots were then washed three times with PBST for 5 min each and developed using an enhanced chemiluminescence reagent using ImageJ software (NIH, Bethesda, MD, USA).

### 2.8. Statistical Analysis

All data are presented as the mean ± standard deviation (SD) of at least three experiments in triplicate. All statistical analyses were conducted using Graph Pad Prism 6.0 software (La Jolla, CA, USA). Data obtained from the aforementioned experiments were analyzed using one-way ANOVA or Student's *t*-test when necessary. A statistically significant difference was indicated as *P* < 0.05 compared with untreated controls.

## 3. Results

### 3.1. Hispidulin Increases TMZ-Induced Cytotoxicity in U87MG Cells

**Figure 1:**
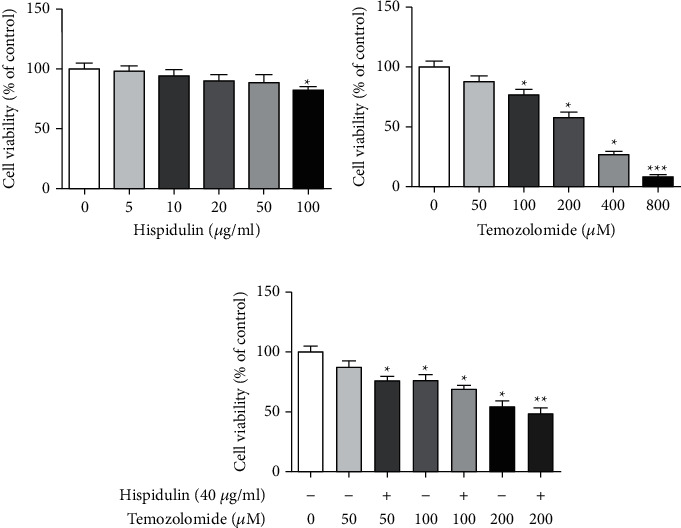
Cytotoxicity of hispidulin, TMZ, or their combination on U87MG cells. (a) Cytotoxicity of hispidulin (5, 10, 20, 40, and 100 *μ*g/ml) on U87MG cells. (b) Cytotoxicity of TMZ (50, 100, 200, 400, and 800 *μ*M) on U87MG cells. (c) Cytotoxicity of hispidulin (40 *μ*g/ml) and temozolomide (50, 100, and 200 *μ*M) on U87MG cells. The data are expressed as the mean ± SD (*n* = 3). ^*∗*^*P* < 0.05, ^*∗∗*^*P* < 0.01, and ^*∗∗∗*^*P* < 0.001 vs. control.

The cytotoxic effects of hispidulin or TMZ on the growth of U87MG cells were examined by the MTT assay. As seen in Figure 1(a), exposure of U87MG cells to hispidulin at the concentrations of (5, 10, 20, 40, and 100 *μ*g/ml) did not have significant cell loss when compared with untreated control cells. Meanwhile, the cell viability of U87MG cells decreased remarkably in a concentration-dependent manner upon TMZ treatment (50, 100, 200, 400, and 800 *μ*M). Due to the modest inhibition of hispidulin (40 *μ*g/ml) and TMZ (50, 100, and 200 *μ*M) on U87MG cell proliferation, we choose their combination to evaluate if hispidulin has the adjuvant effect on TMZ-treated U87MG cells. As expected, treatment with TMZ at the concentration of 50, 100, and 200 *μ*M alone elicited a light drop in viable cells, while coadministration with hispidulin (40 *μ*g/ml) significantly decreased cell viability. This findings preliminarily indicate that additional administration of hispidulin is beneficial to aggravate the cell death of U87MG cells induced by TMZ.

### 3.2. Hispidulin Promotes TMZ-Induced Apoptosis in U87MG Cells

**Figure 2:**
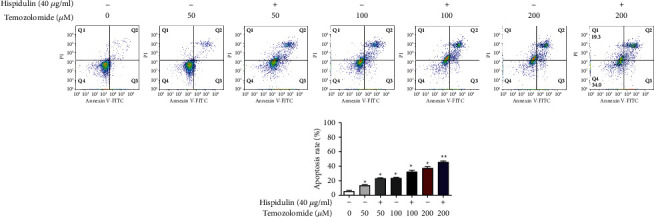
The effect of hispidulin on TMZ-induced apoptosis in U87MG cells evaluated by the flow cytometer. (a) Flow cytometric analysis of annexin-V and propidium iodide (PI) double staining in U87MG cells. (b) Quantification results of apoptosis in U87MG cells. The data are expressed as the mean ± SD (*n* = 3). ^*∗*^*P* < 0.05 and ^*∗∗*^*P* < 0.01 vs. control.

**Figure 3:**
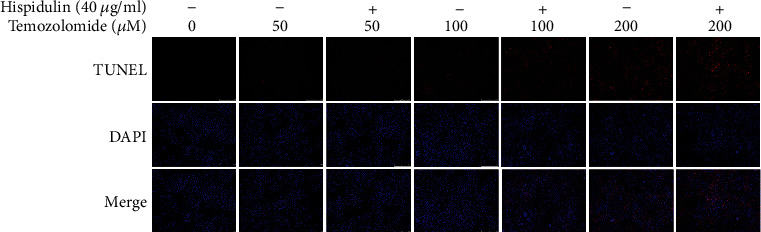
The effects of hesperidin on TMZ-induced apoptosis in U87MG cells evaluated by the TUNEL assay.

Apoptosis detection with annexin V/PI double staining was performed to investigate if additional administration of hispidulin favored the induction of apoptosis in TMZ-treated U87MG cells. TMZ treatment alone resulted in a moderate increase of apoptotic cells in U87MG cells. Intriguingly, the additional administration of hispidulin (40 *μ*g/ml) increased the percentages of total apoptosis of U87MG cells when compared with cells treated with TMZ at each concentration, although the results were not statistically different (*P* > 0.05) (Figures 2(a) and 2(b)). In line with this result, the TUNEL staining assay also demonstrated the enhancement of apoptosis by hispidulin in TMZ-treated U87MG cells (Figure 3). These findings indicate that the additional administration of hispidulin can promote apoptosis in TMZ-treated U87MG cells.

### 3.3. Hispidulin Modulated TMZ-Induced Related Apoptosis Protein Expression in U87MG Cells

**Figure 4:**
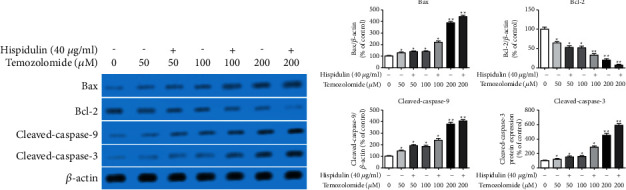
The effects of hesperidin on the change of proapoptosis and antiapoptosis protein expressions in TMZ-treated U87MG cells valuated by the Western blot assay. (a) The effects of hesperidin on the protein expression of Bax, Bcl-2, cleaved-caspase-3, and cleaved-caspase-9 in U87MG cells. (b) Quantification results of the protein expression of Bax, Bcl-2, cleaved-caspase-3, and cleaved-caspase-9 in U87MG cells. The data are expressed as the mean ± SD (*n* = 3). ^*∗*^*P* < 0.05 and ^*∗∗*^*P* < 0.01 vs. control.

We next evaluated the expression change of apoptotic related proteins in TMZ-treated U87MG cells. As seen in Figures 4(a) and 4(b), the expression of Bax, cleaved-caspase-3, and cleaved-caspase-9 proteins was enhanced in U87MG cells in response to TMZ treatment at the concentration of 50, 100, and 200 *μ*M, but Bcl-2 expression was attenuated in the same condition. As anticipated, the presence of hispidulin at 40 *μ*g/ml increased the expression of Bax, cleaved-caspase-3, and cleaved-caspase-9 proteins, as well as decreased Bcl-2 protein expression in TMZ-treated U87MG cells, which suggests the enhancement of TMZ-induced apoptosis by hispidulin. These results suggest that hispidulin has a synergistic effect on TMZ-induced apoptosis in U87MG cells.

### 3.4. Hispidulin Inhibited TMZ-Induced Autophagy in U87MG Cells

**Figure 5:**
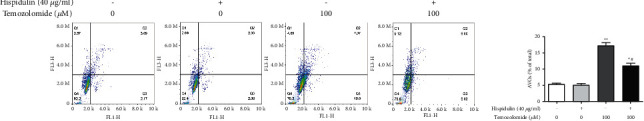
The effects of hesperidin on the change of autophagy in TMZ-treated U87MG cells evaluated by flow cytometric analysis. (a) The effects of hesperidin on the number of AVOs in U87MG cells. (b) Quantification of AVOs detected by flow cytometry with AO staining. The data are expressed as the mean ± SD (*n* = 3). ^*∗*^*P* < 0.05 and ^*∗∗*^*P* < 0.01 vs. control. ^#^*P* < 0.05 vs. TMZ control.

**Figure 6:**
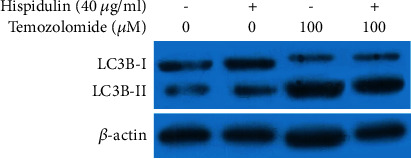
The effects of hesperidin on the change of LC3B-I and LC3B-II protein expressions in TMZ-treated U87MG cells valuated by the Western blot assay.

To determine the role of autophagy in this process, we conducted a flow cytometric analysis to examine the percentage of AVOs in differently treated cells. The result in Figures 5(a) and 5(b) show that hispidulin alone did not exert any effect on autophagy as compared with untreated control cells (*P* > 0.05), while pretreatment with TMZ augmented autophagy (*P* < 0.05). Of note, fewer AVOs were seen in U87MG cells treated with both hispidulin and TMZ. Meanwhile, the expression of LC3B-I and LC3B-II declined significantly in U87MG cells upon the additional administration of hispidulin (*P* < 0.05, Figure 6). These results demonstrate that hispidulin can attenuate TMZ-induced autophagy to promote apoptosis.

## 4. Discussion

The previous investigation has demonstrated that hispidulin potentiates the antitumor activity of TMZ in glioblastoma [16]. However, the mechanism underlying its antitumor role remained fully illustrated. In the present study, we demonstrated for the first time that hispidulin enhanced the antitumor efficacy of TMZ via suppressing TMZ-induced autophagy and subsequently increased apoptotic cell death, suggesting its clinical potential for treating glioblastoma.

First, the MTT assay was adopted to evaluate the effect of hispidulin, TMZ, or their combination on the cell viability of human malignant glioma U87MG cells. TMZ could inhibit the proliferation of U87MG cells in a series of concentrations (100, 200, 400, and 800 *μ*M), which was significant from control cells. Furthermore, almost no change was observed in the U87MG cell following 48 h treatment of hispidulin until 100 *μ*g/ml. Therefore, the doses of 100, 200, and 400 *μ*M of TMZ and 40 *μ*g/ml of hispidulin were selected for use in the following experiments to study the role of hispidulin in TMZ-induced cytotoxicity. Intriguingly, the addition of 40 *μ*g/ml of hispidulin resulted in an enhancement of cell death in cells treated with 100, 200, and 400 *μ*M of TMZ, respectively. These results indicate that hispidulin can promote cell death induced by TMZ in U87MG cells.

Apoptosis is a well-documented cause of cell proliferation inhibition [17], and the ability to induce apoptosis in cancer cells is a key criterion for screening anticancer drugs [18]. Annexin V/PI double staining and TUNEL/DAPI staining assays showed the same tendency as observed in the MTT assay. Coadministration of 40 *μ*g/ml of hispidulin with 100, 200, or 400 *μ*M of TMZ significantly increased the percentage of apoptotic cells.

Bcl-2 family members, Bax (proapoptotic) and Bcl-2 (antiapoptotic), are critical in the regulation of apoptotic cell death [19, 20]. A change of the Bax/Bcl-2 ratio is known to ultimately activate the caspase family of proteins, such as caspase-9 and caspase-3 [18]. The latter is a crucial effector of caspase, which activates PARP cleavage and finally facilitates apoptosis [21]. To further explore the involved molecular mechanisms, western blotting was performed to detect apoptosis-related proteins of the Bcl-2 and caspase family. Consistent with the above findings, we obtained evidence that TMZ treatment resulted in a moderate upregulation of Bax, cleaved-caspase-9, and cleaved-caspase-3 protein expression but downregulation of Bcl-2 protein expression in U87MG cells. However, this tendency was enhanced after combination treatment with 40 *μ*g/ml of hispidulin. These results indicate that hispidulin addition triggered mitochondria-mediated apoptosis by TMZ in U87MG cells.

There are a total of three ways that contribute to the cell death, namely, apoptosis (Type I programmed cell death), autophagy (Type II programmed cell death), and necrosis [22]. Among them, autophagy serves an important role in maintaining cellular homeostasis and also protects against tumor cell death under chemotherapy in some cases [9, 23–25]. Recently, substantial experimental evidence indicates that TMZ-induced autophagy can protect glioma cells from cell death [26]. Blocking this autophagic process can be used as an adjuvant approach for promoting the antitumor efficiency of TMZ against malignant glioma [6, 27]. Given this, we assessed the role of autophagy in different groups. As expected, the result of flow cytometric analysis revealed that more AVOs, a characteristic of autophagy [8], were observed in TMZ-treated U87MG cells. In the same condition, hispidulin was found to suppress TMZ-induced autophagy, as shown by the decrease of AVOs, when compared with TMZ control. Along with the downregulation of Bcl-2 and upregulation of Bax, cleaved-caspase-3, and -9, the levels of LC3B, a critical autophagy marker [28, 29], were also lower compared with the TMZ control group. These results suggest that inhibition of autophagy by hispidulin may promote TMZ to induce apoptosis in U87MG cells.

## 5. Conclusion

In conclusion, the present study demonstrated for the first time that hispidulin could enhance the therapeutic capacity of TMZ *in vitro* by overwhelming TMZ-induced autophagy and increasing apoptosis. These results may provide an advantageous strategy to treat glioma patients with TMZ chemoresistance. However, the more in-depth mechanism involved in this work remains to be advanced in further studies.

## Data Availability

The datasets used and analyzed during the current study are available from the corresponding author on reasonable request.
